# Multidisciplinary practice guidelines for the diagnosis, genetic counseling and treatment of pheochromocytomas and paragangliomas

**DOI:** 10.1007/s12094-021-02622-9

**Published:** 2021-05-06

**Authors:** R. Garcia-Carbonero, F. Matute Teresa, E. Mercader-Cidoncha, M. Mitjavila-Casanovas, M. Robledo, I. Tena, C. Alvarez-Escola, M. Arístegui, M. R. Bella-Cueto, C. Ferrer-Albiach, F. A. Hanzu

**Affiliations:** 1 Medical Oncology Department, Hospital Universitario 12 de Octubre, Instituto de Investigación Sanitaria Hospital 12 de Octubre (imas12), UCM, CNIO, CIBERONC, Avda Cordoba km 5.4, 28041 Madrid, Spain; 2 Radiology Department, Hospital Clínico San Carlos, Madrid, Spain; 3 Endocrine and Metabolic Surgery Unit, General and Digestive Surgery Department, Hospital General Universitario Gregorio Marañón, Madrid, Spain; 4 Nuclear Medicine Department, Hospital Universitario Puerta de Hierro, Majadahonda, Spain; 5 Grupo de Trabajo de Endocrino de la SEMNIM, Madrid, Spain; 6 Hereditary Endocrine Cancer Group, Spanish National Cancer Research Center, Madrid, Spain; 7 Centro de Investigación Biomédica en Red de Enfermedades Raras (CIBERER), Madrid, Spain; 8 Scientific Department, Medica Scientia Innovation Research (MedSIR CORP), Ridgewood, NJ USA; 9 Medical Oncology Department, Hospital Provincial, Castellon, Spain; 10 Neuroendocrinology Unit, Endocrinology and Nutrition Department, Hospital Universitario la Paz, Madrid, Spain; 11 ENT Department, Hospital General Universitario Gregorio Marañón, Madrid, Spain; 12 Pathology Department, Hospital Universitario Parc Taulí, Sabadell, Institut D’Investigació I Innovació Parc Taulí (I3PT), Universitat Autònoma de Barcelona, Sabadell, Spain; 13 Radiation Oncology Department, Hospital Provincial Castellón, Castellón, Spain; 14 Endocrinology and Nutrition Department, Hospital Clinic Barcelona, University of Barcelona, IDIBAPS, Barcelona, Spain

**Keywords:** Pheochromocytoma, Paraganglioma, Diagnosis, Treatment, Genetic counseling, Multidisciplinary, Guidelines

## Abstract

Pheochromocytomas and paragangliomas (PPGLs) are rare neuroendocrine tumors that arise from chromaffin cells of the adrenal medulla and the sympathetic/parasympathetic neural ganglia, respectively. The heterogeneity in its etiology makes PPGL diagnosis and treatment very complex. The aim of this article was to provide practical clinical guidelines for the diagnosis and treatment of PPGLs from a multidisciplinary perspective, with the involvement of the Spanish Societies of Endocrinology and Nutrition (SEEN), Medical Oncology (SEOM), Medical Radiology (SERAM), Nuclear Medicine and Molecular Imaging (SEMNIM), Otorhinolaryngology (SEORL), Pathology (SEAP), Radiation Oncology (SEOR), Surgery (AEC) and the Spanish National Cancer Research Center (CNIO). We will review the following topics: epidemiology; anatomy, pathology and molecular pathways; clinical presentation; hereditary predisposition syndromes and genetic counseling and testing; diagnostic procedures, including biochemical testing and imaging studies; treatment including catecholamine blockade, surgery, radiotherapy and radiometabolic therapy, systemic therapy, local ablative therapy and supportive care. Finally, we will provide follow-up recommendations.

## Introduction

Pheochromocytomas (PCCs) and paragangliomas (PGLs)—hereinafter PPGL to include both entities—are rare neuroendocrine tumors (NETs) that arise from chromaffin cells of the adrenal medulla and the sympathetic/parasympathetic neural ganglia, respectively. PPGLs often secrete catecholamines (CMNs) that can mimic a wide range of medical disorders and may be lethal if misdiagnosed or improperly handled. PPGLs are also characterized by a very heterogeneous natural history and a low to moderate but unpredictable ability to metastasize. Surgical resection may be challenging, including adequate timing and perioperative medical management, and optimal treatment of advanced disease is controversial. Over one-third of PPGLs are inherited, and adequate genetic counseling is key to implementing screening strategies and tailoring therapy. All these factors make PPGL diagnosis and treatment very complex, and clinical experience is difficult to achieve due to their low incidence. In this context, the aim of this article was to provide practical clinical guidelines for the diagnosis and treatment of PPGLs from a multidisciplinary perspective. Experts from the national societies of the different disciplines involved participated in the elaboration of these guidelines, including clinical specialists from the Spanish Societies of Endocrinology and Nutrition (SEEN), Medical Oncology (SEOM), Medical Radiology (SERAM), Nuclear Medicine and Molecular Imaging (SEMNIM), Otorhinolaryngology (SEORL), Pathology (SEAP), Radiation Oncology (SEOR), and Surgery (AEC), and geneticists from the Spanish National Cancer Research Center (CNIO).

## Epidemiology

The joint annual incidence of PPGL is estimated to be 2–8 cases per million inhabitants. A recent study from Canada disclosed an annual incidence of 6.6 cases per million inhabitants, half of them corresponding to pheochromocytomas and 37% of them to head and neck paragangliomas [1], most of them known to be parasympathetic. Head and neck paragangliomas have geographic variations as a function of altitude [2]. In another study from Holland [3], the annual incidence of PCCs and of sympathetic PGLs were 4.6 and 1.1 cases per million inhabitants, respectively, which had increased compared to previous years likely due to improved diagnostic techniques and clinical awareness.

The distribution by gender does not show significant differences, although a greater incidence in females has been observed for vagal and jugulotympanic PGLs [4], and in high-altitude PGLs [2]. PPGLs are commonly diagnosed within the 4th and 6th decades, although these neoplasms can occur over a wide age range. They appear at a younger age when they occur as part of a hereditary syndrome [5]. At pediatric ages, extra-adrenal PGLs account for more than two-third of the cases, and four of five cases are associated with a hereditary form of the disease [6, 7].

Bilateral or multiple forms are mostly associated with hereditary syndromes, but an advanced age at diagnosis or the absence of family history does not exclude the possibility of carrying a germline mutation. In fact, between 14 and 24% of clinically sporadic tumors may also be due to a germline mutation [8, 9].

The rate of metastatic disease (mPPGL) ranges from less than 1% to 79%, depending upon tumor site and size, age at diagnosis and genotype [10, 11]. Although some features included size greater than 5 cm, extra-adrenal primary tumor site, or high levels of plasma 3-metoxitiramine (3-MT) [12] provide useful information to assess the risk of metastasis, the presence of mutations in the succinate dehydrogenase complex iron sulfur subunit B (*SDHB*^Mut^) is the only universally accepted criterion associated with a high risk of distant disease, both at diagnosis or during follow-up, ranging from 20 to 70% in different patient cohorts [11–14]. Recent data also suggest a higher metastatic risk in patients with mutations in other genes involved in the Krebs cycle [15].

Overall, the prognosis of PPGL is heterogeneous. Goffredo et al. analyzed 508 PPGL patients from 18 US registries (time frame 1988–2009) and reported a 5-year overall survival (OS) rate of 58% for metastatic PCCs and 80% for metastatic PGLs [16]. More recently, a retrospective study of 169 patients from 18 European centers (time frame 1998–2010) by Hescot et al. [14] reported a global 5-year OS rate of 62% and a median OS of 6.7 years for mPPGL.

## Anatomy, pathology and molecular pathways

### Anatomy

PCC (adrenal) and PGL (extra-adrenal) are neoplasms that originate from chromaffin cells of the autonomic nervous system, derived from the neural crest, and can be classified as either sympathetic or parasympathetic. The sympathetic system is distributed through the paraganglia of the prevertebral and paravertebral axes, reaching the abdominal organs and innervating the urogenital system, and it includes the adrenal medulla, which is considered the greatest paraganglion. The parasympathetic system is distributed through the head and neck and mediastinum, following the territories of the glossopharyngeal (carotid and tympanic paraganglia) and vagus (jugular, upper and lower laryngeal, subclavian and aortopulmonary) nerves [17]. These neoplasms are also grouped into cervicocephalic, thoracic and abdominal PPGLs [18].

### Histopathological features

The characteristic microscopic feature of PPGLs is the proliferation of polygonal chromaffin cells distributed in nests (zellballen, in German), surrounded by a fine capillary network and sustentacular cells, easily identifiable through immunohistochemical (IHC) detection of S-100. This pattern is more evident in parasympathetic PGLs. Chromogranin A (CgA) and synaptophysin, but not cytokeratins, are expressed in tumor cells. Tyrosine hydroxylase is also expressed in PCCs and sympathetic PGLs but not in parasympathetic PGLs. IHC studies with Ki67 (Mib-1) are also recommended to estimate the cellular proliferation index [5].

### Stratification of risk and staging

All PPGLs are potentially metastatic; therefore, benign vs malignant discrimination was eliminated in the last World Health Organization (WHO) classification for endocrine tumors [5]. Several scoring systems have been established to estimate the metastatic risk of these tumors. The Pheochromocytoma of the Adrenal Gland Scaled Score (PASS) [19], applicable only to PCCs, is based solely on morphological criteria, some of which may be too subjective to evaluate (Table 1). The Grading of Adrenal Pheochromocytoma and Paraganglioma (GAPP) scale [20] is applicable to both PCCs and PGLs and combines morphological, IHC and analytical findings (Table 1).

**Table 1:** Pheochromocytoma and paraganglioma risk stratification systems

Parameter	Score
Pheochromocytoma of the adrenal gland scaled score (PASS)	
Large nests or diffuse growth (> 10% of tumor volume)s	2
Central or confluent tumor necrosis	2
High cellularity	2
Cellular monotony	2
Tumor cell spindling	2
Mitotic figures > 3/10 high-power field	2
Atypical mitotic figure(s)	2
Extension into adipose tissue	2
Vascular invasion	1
Capsular invasion	1
Marked nuclear pleomorphism	1
Nuclear hyperchromasia	1
Total	20*
*A score > 4 implies a higher risk of metastases	

Grading of adrenal pheochromocytoma and paraganglioma (GAPP)	
Architectural pattern	
Regular cell nest	0
Large and irregular cell nest	1
Pseudorosette	1
Cellularity	
Low (< 150 cells/U)	0
Moderate (150–250 cells/U)	1
High (> 250 cells/U)	2
Presence of comedo-type necrosis	2
Presence of vascular or capsular invasion	1
Ki67 labeling index (%)	
< 1	0
1–3	1
> 3	2
Catecholamine type	
Adrenergic type (± noradrenaline)	0
Noradrenergic type (noradrenaline ± dopamine	1
Nonfunctioning	0
Total maximum score	10

Grade	Score	Metastatic rate (%)	5-year survival (%)
Well differentiated	0–2	3.6	100
Moderately differentiated	3–6	60	66.8
Poorly differentiated	7–10	88.2	22.4

Composite pheochromocytoma/paraganglioma prognostic score (COPP)	
Focal or confluent tumor necrosis	5
Loss of S100 expression	2
Vascular invasion	1
Loss of SDHB expression	1
Size > 7 cm	1
A score greater than or equal to 3 means a high metastatic risk	

IHC studies of SDH components are indicated and are especially useful in the study of subunit B of SDH (SDHB), as the loss of expression indicates the presence of an *SDH* germline mutation and an increased risk of aggressive behavior [21, 22]. In fact, SDHB loss of expression is included in the most recent risk stratification score, COPPS (Composite Pheochromocytoma/paraganglioma Prognostic Score) (Table 1) [23]. The eighth edition of the American Joint Committee on Cancer (AJCC) staging system [24] includes a new chapter to address PCC and sympathetic PGL (Table 2), but not parasympathetic PGL, given its low risk of malignant behavior (~ 5%) [25, 26].

**Table 2:** Staging of pheochromocytomas and sympathetic paragangliomas according to the American Joint Committee on Cancer (AJCC)—Cancer Staging 8th edition

Definition of primary tumor (T)			
T category		T criteria	
TX		Primary tumor cannot be assesed	
T1		Pheochromocytoma < 5 cm in greatest dimension, no extra-adrenal invasion	
T2		Pheochromocytoma ≥ 5 cm or sympathetic Paraganglioma of any size, no extra-adrenal invasion	
T3		Tumor of any size with invasion into surrounding tissues (e.g., liver, pancreas, spleen, kidneys)	
Definition of regional lymph node (N)			
N category		N criteria	
NX		Regional lymph nodes cannot be assessed	
N0		No lymph node metastasis	
N1		Regional lymph node metastasis	
Definition of distant metastasis (M)			
M0		No distant metastasis	
M1		Distant metastasis	
M1a		Distant metastasis to only bone	
M1b		Distant metastasis to only distant lymph nodes/liver or lung	
M1c		Distant metastasis to bone plus multiple other sites	
AJCC prognostic stage groups			
T	N	M	Stage
T1	N0	M0	I
T2	N0	M0	II
T1	N1	M0	III
T2	N1	M0	III
T3	Any N	M0	III
Any T	Any N	M1	IV

### Molecular basis of PPGL

A total of 30–50% of PPGLs occur in the context of a hereditary syndrome [27–30]. The most common hereditary syndromes are those derived from germline mutations in genes encoding the different subunits of SDH (type 1: *SDHD*; type 2: *SDHAF2*; type 3: *SDHC*; type 4: *SDHB*; type 5: *SDHA*; including Carney-Stratakis syndrome) (15–20%), Von Hippel-Lindau (VHL) syndrome due to mutations in the *VHL* gene (9%), multiple endocrine neoplasia-2 (MEN2) syndrome due to mutations in the *RET* proto-oncogene (5%) and neurofibromatosis type 1 (NF-1) syndrome due to mutations in the *NF1* gene *(2%)*. Less frequent familial forms (< 1–2%) are caused by mutations in the transmembrane protein 127 (*TMEM127*), MYC-associated factor X (*MAX*), fumarate hydratase (*FH*), multiple endocrine neoplasia type 1 (*MEN1*), egg-laying-defective nine (egl-9) family hypoxia-inducible factor 1 gene (*EGLN1*), egl-9 family hypoxia-inducible factor 2 (*EGLN2*), malate dehydrogenase 2 (*MDH2*), kinesin family member 1B (*KIF1B*) genes [31–34], solute carrier family 25 Member 11 (*SLC25A11*) and dihydrolipoamide *S*-succinyltransferase (*DLST*) [35, 36]. Susceptibility genes and familial PPGL syndromes have been comprehensively reviewed recently [37], and their main features are summarized in Table 3.

**Table 3:** PPGL susceptibility genes

Gen	Syndrome	Biochemical profile	Year of discovery	Gene role	Clinical presentation	Mutation type	Cluster	Inheritance	References
*NF1*	Neurofibromatosis Type 1	Adrenergic	1990	TSG: ↓ cell proliferation by blocking RAS/RAF/MAPK and PI3K/AKT/mTOR pathways	Hallmark signs: Café-au-lait Macules *(99% within first year of llife)*, Skinfold freckling (Crowe's sign), Cutaneous neurofribromas, lysch nodules, optic glioma Xanthogranuloma, melanoma (0.1–5.4%), skeletal manifestations (such as scoliosis or macrocephaly), astrocytoma, PPGL, GIST, malignant schwannoma, juvenile myelomonocytic leukemia	G	2	AD	[134]
*RET*	MEN 2	Adrenergic	1993	Proto-oncogene: encodes TKR; which once bound to GTNF activates the RAS/RAF/ERK-signaling pathway, leading to cell proliferation and invasiveness	PCC 50–100% MTC, PCC, HPT, cutaneous amyloidosis, Hirschsprung disease, mucosal neuroma, dysmorphic and, marphanoid features	G	2	AD	[134]
*VHL*	von Hippel-Lindau	Noradrenergic	1993	TSG: ↑HIF2A degradation under HYPOXIA conditions	PPGL 10–20%. Hemangioblastoma (cerebellar, spinal cord, retina), RCC, pNET, pancreatic cysts, yolk sac tumor	G	1	AD	[134]
*MEN1*	MEN 1	Adrenergic	1993	TSG: Regulates transcription, stabilizes genome y ↓ cell proliferation	Parathyroid adenoma, pNET, gastrinoma, pituitary adenomas, adrenal tumor, other carcinoids, lipoma, angiofibroma, meningioma PCC < 1%	G	2	AD	[134]
*SDHD*	Familial PPGL linked to SDHD	Noradrenergic	2000	TSG: encodes SDH that catalyzes the oxidation of succinate to fumarate in the TCA cycle. Increase in succinate leads to stabilization of HIF	↑Penetrance PPGL (> 80%), GIST, pituitary adenomas	G	1	AD***	[134]
*SDHC*	Familial PPGL linked to SDHC		2000		↓↓Penetrance PPGL; other tumor: pituitary adenoma; GIST	G	1	AD	[135]
*SDHB*	Familial PPGL linked to SDHB		2001		Malignant PPGL, penetrance ~ 16–22 and 44%, at 50, 60 and 80 years respectively, RCC (4.2%), GIST, pituitary adenomas	G	1	AD	[134]
*SDHAF2*	Familial PPGL linked to SDHAF2		2009		Unknown penetrance	G	1	AD***	[136]
*SDHA*	Familial PPGL linked to SDHA		2010		↓Penetrance PPGL, GIST	G	1	AD	[137]
*TMEM127*		Adrenergic	2010	TSG: ↓ cell proliferation by blocking PI3K/AKT/mTOR pathways	↓↓Penetrance PPGL; other tumors: RCC	S	2	N/A	[31]
*IDH1*		Noradrenergic	2010	TSG: catalyzes the oxidative decarboxylation of isocitrate in the TCA cycle. Increase in α-ketoglutarate leads to stabilization of HIF	↓↓Penetrance PPGL	S	1	N/A	[138]
*IDH2*			2010			S	1	N/A	[139]
*MAX*		Noradrenergic Adrenergic	2011	TSG: ↓ cell proliferation, regulator of differentiation ↑ apoptosis	Mainly PCC	S	2	AD; paternal transmission	[140]
*FH*	HLRCC	Noradrenergic	2012	TSG; encodes FH that catalyzes the reversible hydration of fumarate to l-malate in the TCA cycle. Increase in fumarate leads to stabilization of HIF	Multifocal PPGL, metastatic, associated HLRCC or MCUL	G	1	AD	[33, 141]
*HIF2A or EPAS1*	Pacak-Zhuang	Noradrenergic	2012	Oncogene; encodes EPAS1; transcription factor related to oxygen-level responses and activated in hypoxic conditions	Triad of PPGLs, polycythemia, and somatostatinoma. Ocular abnormalities occur in 70%	S/M	1	N/A	[142–145]
*H-RAS*		Adrenergic	2013	Proto-oncogene; encodes H-RAS, which once bound to GTP activates the RAS/RAF/ERK-signaling pathway, leading to cell proliferation	Mainly single PCC (Caucasian population), sporadic, mainly benign	S	2	N/A	[146, 147]
*H3F3A*		Unknown	2013	Encodes the histone H3.3 protein that has an essential role in maintaining genome integrity during mammalian development	Giant cell tumors of the bones (GCT), PCCs, bladder and periaortic PPGL	S/M	*	N/A	[148]
*EGLN2*		Noradrenergic	2015	TSG; encodes PHD1, an enzyme that, in normal oxygen conditions, hydroxylates specific proline residues of the HIF-α subunits for posterior degradation in the proteasome	Polycythemia associated with recurrent PPGLs, and normal or mild elevated EPO	G	1	**	[149]
*MDH2*		Noradrenergic	2015	TSG; encodes MDH2, which catalyzes the reversible oxidation of malate to oxaloacetate in the TCA cycle Increase in malate, fumarate and succinate leads to stabilization of HIF	Multiple PGLs, metastatic	G	1	AD	[150]
*ATRX*			2015	Encodes a chromatin remodeling protein that regulates the nuclear matrix and chromatin association	Clinically more aggressive and metastatic PGL	S	*	N/A	[151]
*CSDE1*		Noradrenergic	2017	TSG. Involved in normal development through messenger RNA stability internal initiation of translation, and cell-type-specific apoptosis. Promotes and represses the translation of RNAs and also increases and decreases the abundance of RNAs	Sporadic, metastatic, recurrent PPGL	S	3	N/A	[34]
*MAML3*		Noradrenergic	2017	Oncogene. Encodes a transcriptional coactivator for NOTCH. In PPGLs, with a hypomethylated profile ⟶ mRNA overexpression of the target gene involved in Wnt receptor and Hedgehog signaling pathways	Sporadic, recurrent PGL. New prognostic factor of poor outcome	F	3	N/A	[34]
*IRP1*		Noradrenergic	2018	TSG; encodes IRP1, which controls cellular iron metabolism and negatively regulates HIF2α mRNA translation under iron-deficient conditions. Deficiency of IRP1 protein increases HIF2α	Sporadic, adrenal PCC	S	1	N/A	[152]
*SLC25A11*		Noradrenergic	2018	TSG: encodes the mitochondrial 2-oxoglutarate/malate carrier in the TCA cycle leading to stabilization of HIF	Malignant PPGL, HNPGL	G	1	AD	[36]
*DLST*		Noradrenergic	2019	TSG: encodes the E2 subunit of the mitochondrial αKG dehydrogenase (OGDH). Depletion of any of the OGDH complex subunits leads to impaired enzymatic activity, a-ketoglutarate accumulation and stabilization of HIF	Recurrent multiple PPGLs, malignancy also described, pituitary adenoma, uterine carcinoma also described PPGL >> PCC	G	1	AD	[35]

*AD* Autosomal Dominant, *AKT* serine/threonine kinase, *ATRX* chromatin remodeler ATRX, *CRG* growth regulatory factors, *CSDE1* coldshock domain containing E1, *DLST* Dihydrolipoamide S-Succinyltransferase, *EGLN1/2* egl nine homolog 1 and 2, *EPAS1* PAS domain-containing protein 1, *EPO* erythropoietin, *ERK* extracellular mitogen-activated protein kinase 1, *F* fusion, *FH* fumarate hydratase, *GTNF* glial cell line‐derived neurotrophic factor, *HNPGL* head and neck paraganglioma, *G* germline, *GTC* giant cell tumor of the bone, *H3F3A* H3 histone family member 3A, *HIF2α* hypoxia-inducible factor 2 alpha, *HIF2A* hypoxia-inducible factor 2 alpha, *HLRCC* leiomyomatosis and renal cell cancer, *H-RAS* HRAS proto-oncogene, *IDH1/2* isocitrate dehydrogenase 1 and 2, *IRP1* iron regulatory protein, *M* mosaicism, *MCUL* multiple cutaneous leiomyomatosis, *MDH1/2* malate dehydrogenase type 1 and 2, *MAML3* coactivator 3 mastermind-like, *MAPK*, mitogen-activated protein kinase; *MAX,* myc-associated factor X gene; *Men1*, multiple endocrine neoplasia 1; *MEK*, mitogen-activated protein kinase; *mRNA*, messenger ribonucleic acid; *mTOR*, mammalian target of rapamycin; *N/A*, Not Applicable in the setting of somatic mutations; *NETs*, neuroendocrine tumors; *NF1*, neurofibromin 1; *PCC*, pheochromocytoma; *PGLs*, paraganglioma; *PHD1/2*, prolyl hydroxylase 1 and 2; *PI3K*, phosphatidyl-inositol-3-kinase; *PPGL*, pheochromocytoma-paraganglioma; *RCC*, renal cell carcinoma; S, somatic; *SLC25A11,* Solute Carrier Family 25 Member 11; *SDH*, succinate dehydrogenase subunits *A/B/C/D*; *SDHAF2*, succinate dehydrogenase complex assembly factor 2; *TCA*, tricarboxylic acid, *TFG*, transcription factors genes; *TKR*, tyrosine kinase receptor; *TMEM127*, transmembrane protein 127; *TSG*, tumor suppressor gene; *VHL*, von Hippel Lindau

*Not classified by clusters, **Unknown, ***maternal imprinting

### Molecular classification of PPGL

The genomic characteristics of PCCs and PGLs allow us to distinguish among three groups or main *clusters*. Cluster 1 groups tumors with germinal or somatic mutations in genes related to the Krebs cycle (*SDHA, SDHAF2, SDHB, SDHC, SDHD, FH, MDH2, GOT2, IDH1*, *SLC23A11*, etc.), in *EPAS1* and in *VHL*. The presence of mutations in these genes leads directly or indirectly to HIF1a and HIF2a stabilization and, therefore, to a situation of pseudohypoxia, which causes an increase in angiogenesis and cell proliferation. PPGLs associated with mutations in genes of the Krebs cycle show a characteristic pattern of higher overall methylation, known as the *CpG island methylator phenotype* (CIMP). The level of CIMP is higher among *SDHB*^Mut^ tumors. This phenotype leads to expression deregulation of genes involved in neuroendocrine differentiation or in the epithelial-mesenchymal transition process, findings that could explain the increased risk of metastasis among patients with *SDHB*^Mut^ tumors. In a recent study higher DNA methylation levels were found in metastatic SDHB-PPGLs as compared to SDHB-PPGLs without metastasis, and this included de novo methylation of protocadherins (PCDH). Furthermore, in vitro assays suggested PCDHGC3 as a putative suppressor gene and a potential biomarker to identify patients with SDHB-mutated cancer at high risk of metastasis [38]. Cluster 2 groups tumors with mutations in *NF1, RET, HRAS* and *TMEM127* genes*,* which activate the MAP kinase signaling pathway [39]. The Cancer Genome Atlas (TCGA) project has identified a third group or cluster related to alterations in the Wnt pathway [34], which also seems to be associated with an increased risk of developing metastatic disease (Table 3).

## Clinical presentation

The clinical presentation of PPGLs is extremely variable and depends on the anatomical location, tumor size and extent of locoregional or distant involvement; the secretion or not of catecholamines (CMNs), including type, amount and pattern of secretion (adrenaline/epinephrine: A/E; noradrenaline/norepinephrine: NA/NE, and dopamine: DA); the hereditary or sporadic nature; the malignancy potential; and the time elapsed from initiation of symptoms to diagnosis.

PPGLs arising from sympathetic paraganglia are characterized by adrenergic and noradrenergic symptoms, such as the classic triad of palpitations, headache and diaphoresis or tremor, facial pallor and dyspnea. However, the predominant symptom remains severe, variable hypertension (65%), with target tissue damage such as hypertrophic or dilated cardiomyopathy (including Tako Tsubo idiopathic cardiomyopathy-like forms), potentially leading to fatal cardiac events, arrhythmias, myocardial infarction, congestive heart failure and chronic lung disease. Hemodynamic instability due to alterations in sympathetic vascular tone and orthostatic hypotension has also been described [12, 40–47]. Other symptoms of CMN excess include increased basal metabolism, weight loss, sweating, heat intolerance, altered glucose homeostasis resulting in type 2 diabetes mellitus, polyuria, polydipsia, constipation, ischemic colitis, altered vision, increased erythrocyte sedimentation rate and leukocytosis, psychiatric symptoms, and, rarely, hypercalcemia and polycythemia.

Symptoms are variable in duration and frequency and can be spontaneous or induced by various stimuli, such as food with high tyramine content (chocolate, coffee, smoked meat, cheese, red wine), sustained physical exercise (sometimes during urination in bladder PGLs), delivery, trauma, the induction of anesthesia, invasive diagnostic or therapeutic procedures, surgery, tumor biopsy or fine-needle aspiration and some medications (DA-2 antagonists, β-adrenergic blockers, sympathomimetics, opioids, tricyclic antidepressants, serotonin reuptake inhibitors, monoamine oxidase (MAO) inhibitors, corticosteroids, peptides or neuromuscular blocking agents) [12, 47]. Patients with A/NA PPGLs may be asymptomatic due to early diagnosis in the setting of abdominal imaging performed for other reasons (5% of adrenal incidentalomas) or screening procedures in at-risk family members [12, 45, 47]. Early case detection reduces cardiovascular morbidity and mortality due to chronic untreated CMN secretion [12, 43, 44, 47].

PGLs distributed along the parasympathetic chains of the head and neck (glossopharyngeal and vagal nerves) tend to be silent or pseudo-silent tumors (up to 15% of PPGLs). Usually, they do not produce CMNs, have a low metastatic potential, present as a head and neck mass with symptoms related to tumor bulk and local compression, or are incidentally discovered on imaging studies done for other purposes [12, 45, 47].

Recently, PPGLs have been classified based on translational clinical-biochemical-gene mutation cluster features into five groups: silent, biochemically pseudo-silent, noradrenergic, adrenergic and dopaminergic phenotypes [37, 48].

PPGLs associated with hereditary predisposition syndromes are more likely to be recurrent, multifocal and bilateral; occur at a younger age [12, 45, 47]; have a higher malignancy potential; and are associated with genetically driven comorbidities. Table 4 summarizes the main screening indications for PPGLs.

**Table 4:** Screening indications for PPGLs

Paroxysmal episodes of palpitations, headaches, diaphoresis, pallor and hypertension
Unexplainable variability in blood pressure
Severe treatment-resistant blood pressure
Paradoxical blood pressure response to drugs, food, anesthesia, surgery
Orthostatic hypotension in a hypertensive patient
New-onset diabetes mellitus in a young lean hypertensive patient
Adrenal incidentaloma
Genetic predisposition for hereditary PPGL

*PPGL* pheochromocytomas and paragangliomas

Clinical severity and prognosis are marked by the malignancy potential of PPGLs and the risk of aggressive metastatic evolution. Malignancy, defined as the presence of metastasis in non-chromaffin tissues [49], has a prevalence of 10% in PCCs and reaches 35–40% in PGLs [12, 45, 47], with symptoms and disease burden depending on the affected tissue. Translational risk scores are currently focusing on the identification of tumors with an aggressive outcome.

## Hereditary predisposition syndromes and genetic counseling and testing

A hereditary form of PPGL should always be ruled out following the diagnoses of PPGL. Syndromic PPGL is strongly suspected in an individual with multiple, multifocal, recurrent, early onset of the disease and/or a family history of PPGL or related tumors (see predisposition syndromes associated with germinal mutations in Table 3). There are currently over 22 susceptibility genes identified. Among them, genes related to syndromic presentation drive nearly half of the cases. PPGLs present the highest rate of germline susceptibility in cancer genetics, at almost 40% [50–52]. These include genes encoding neurofibromin 1 (*NF1*), *RET*, *VHL*, menin (*MEN1*), SDH complex (*SDHx: SDHA, SDHB, SDHC, SDHD*), SDH complex assembly factor 2 (*SDHAF2*), *TMEM127*, *MAX, FH*, hypoxia-inducible factor 2A (*EPAS1/HIF2A*), *EGLN1/PHD2*, *SLC25A11* and *DLST*. These genes are inherited in an autosomal-dominant manner. However, pathogenic variants in *SDHD* cause disease only when the pathogenic variant is inherited from the father. Similarly, SDHAF2 and possibly *MAX* follow an autosomal dominant inheritance, modified by maternal imprinting. Taking into account this genetic heterogeneity, next-generation sequencing (NGS) has been currently established as the new standard screening tool for genetic testing in patients with PPGL. Susceptibility genes and familial PPGL syndromes have been comprehensively reviewed recently [37], and their main features are summarized in Table 3.

The genotype–phenotype correlation is a useful tool to assess the clinical outcome of patients. These include tumor location and CMN secretion profile, as well as the presence of metachronous tumors, aggressive behavior and overall prognosis. Regarding the biochemical profile, genes involved in the pseudohypoxia pathway (Cluster 1) typically are associated with NE and its main metabolite, normetanephrine (NMN) [53, 54] and tumors are commonly located outside the adrenal glands [53, 54]. When located in the head and neck region (HNPGLs), PPGLs have been traditionally classified as biochemically “silent” tumors [55, 56]. However, HNPGLs can also produce high levels of DA and its main metabolite, 3-MT, with normal or near-normal levels of NE/NMN [57, 58]. Elevated levels of DA/3MT/NE have been reported in approximately 65% of patients with *SDHx*^Mut^ tumors [58, 59]. Hereditary forms related to Cluster 1 genes often present with variable expressivity and incomplete penetrance. For instance, patients with a driver mutation in *SDHA*/C usually lack a positive family history and rarely present with more than one tumor. Finally, regarding recurrence or aggressive disease, almost 90% of patients with metastatic PGLs present a *SDHA/B*^Mut^ or *FH*^Mut^ tumor [33, 60–62]. On the other hand, genes involved in the kinase signaling group pathway (Cluster 2) commonly present with adrenal tumors that produce either purely elevated E or its main metabolite, metanephrine (MN) [54], or both E/MN and NE/NMN. This hereditary form often presents with higher expressivity, and complete penetrance occurs more frequently.

If the pathogenic variant has been identified in an affected family member, prenatal testing for a pregnancy at increased risk and preimplantation genetic diagnosis could be considered in certain syndromes, such as VHL disease. Furthermore, genetic identification provides valuable information for the establishment of a treatment plan and for appropriate guidance for follow-up surveillance.

## Diagnostic procedures

**Fig. 1:**
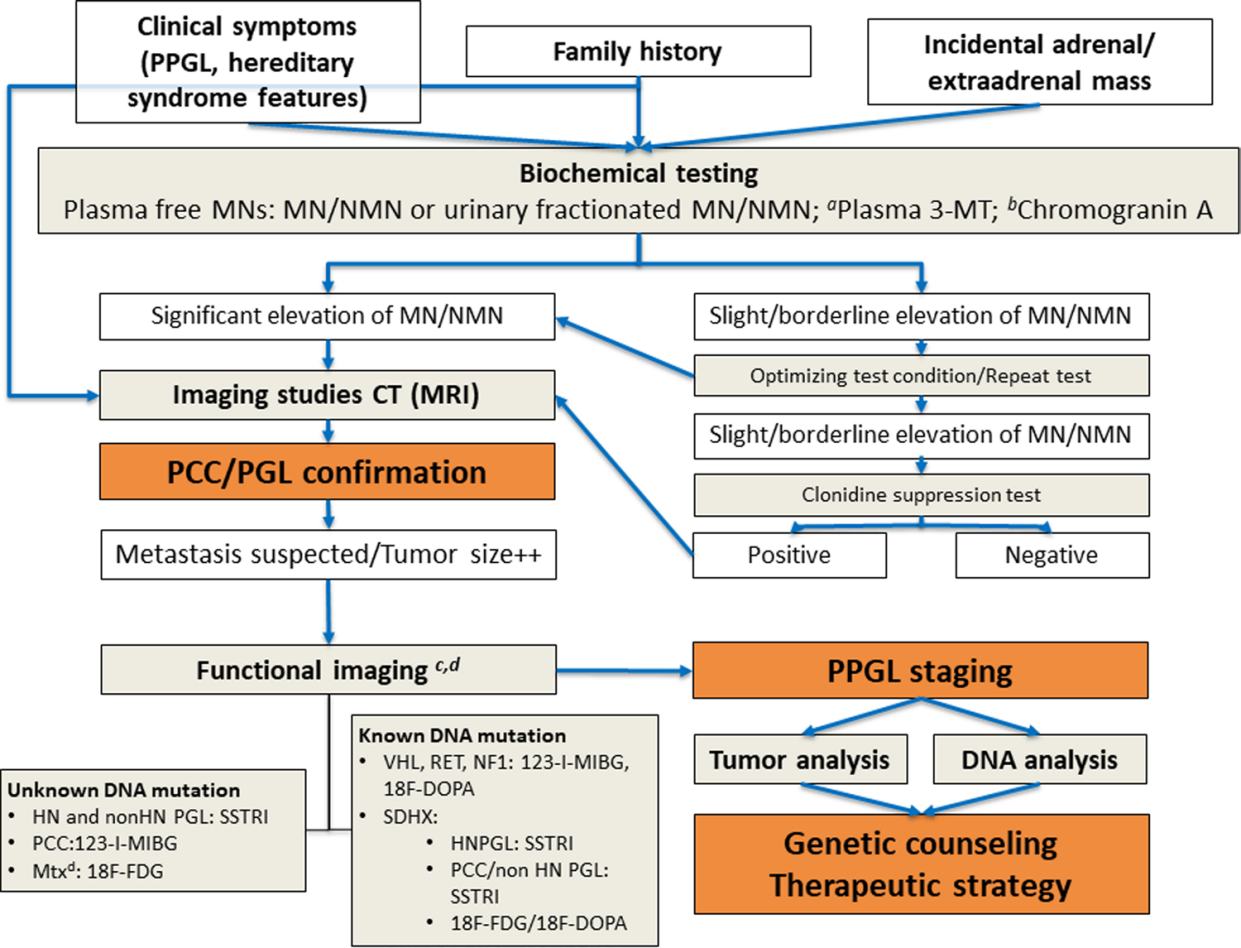
Diagnostic algorithm for PPGLs. *CT* computed tomography, *FDG* fluorodeoxyglucose, *HNPGL* head and neck paraganglioma, *MN* metanephrine, *Mtx* metastasis, *NMN* normetanephrine, *MIBG* metaiodobenzylguanidine, *3-MT* 3-methoxytyramine, *MRI* magnetic resonance imaging, *PCC* pheochromocytomas, *PGL* paragangliomas, *PPGL* pheochromocytomas and paragangliomas, *SSTRI* somatostatin receptor imaging, *VHL* von Hippel-Lindau. **a** Plasma 3-MT: only in high clinical suspicion of dopamine-secreting tumors/hereditary syndromes associated with HNPGL. **b** Chromogranin A: nonspecific neuroendocrine tumor marker that may be considered if high clinical suspicion of silent PPGLs. **c** Recommended at diagnosis only in cases of high suspicion of metastasis, particular if there is family history or silent tumor. **d** 123-I-MIBG versus ^111^In/^99^mTc/^68^ Ga SSTRI, is recommended before MIBG versus radionuclide-SSTR analogs treatment

The diagnosis of PPGLs includes clinical suspicion, biochemical hormonal detection of excess CMN secretion, imaging studies for tumor localization and staging, genetic screening and, if a genetic germline mutation is confirmed, additional diagnostic procedures for genetic syndromic features as appropriate. PPGLs diagnosed as incidental masses in imaging studies require the same approach. A proposed diagnostic algorithm is provided in Fig. 1.

### Biochemical testing

The biochemical diagnosis and follow-up of PPGLs rely on the quantification of CMN metabolites, such as plasma-free NMN, MN, and 3-MT or urinary fractionated MNs (e.g., MN, NMN). CMN measurement is less informative. Reference intervals (supine position; children) and standard preanalytical conditions (supine position for plasma determination, diet and medication interferences for urine testing) have to be carefully followed. Biochemical testing for PPGLs should be performed before imaging studies. Prior to the 24-h urine and plasma CMN metabolite determination, a 3-day diet without ca**f**feine, black tea, nicotine, alcohol, bananas, cheese, almonds, nuts, chocolate, eggs, or vanilla is recommended. Some drugs, such as MAO inhibitors, ephedrine, cocaine, tricyclic antidepressants, serotonin reuptake inhibitors, morphine, amoxicillin, levodopa, sulfasalazine, acetaminophen, methyldopa and buspirone, can also cause false-positive results and should be avoided if possible [12, 63–65].

Guidelines accept both plasma-free and urinary MNs for the screening of PCCs and PGLs, with both determinations being considered to have similar sensitivities (97%) and specificities (91%) [12]. Some reports showed a better sensitivity (99% versus 80%) and a lower specificity (85% versus 98%) for plasma versus urinary MNs. Urinary creatinine is required to normalize urinary metanephrine excretion to renal function (metanephrine-to-creatinine ratio). Recent results, conversely, reported that plasma-free MNs in the supine position have higher specificity than 24-h urinary fractionated MNs (95% versus 90%), with the highest accuracy (95%) for liquid chromatography–mass spectrometry (LC–MS) methods compared with immunoassays [65–67]. LC–MS or electrochemical detection [liquid chromatography electron capture dissociation (LC-ECD)] is considered the gold standard, avoiding drug interference [12, 63–66].

Plasma levels of NMNs, MNs or 3-MT more than twice the upper cutoff value of the reference interval indicate a high probability of PPGL, and further imaging studies are recommended. Combined increases in two or more metabolites also suggest a high probability of a PPGL.

In the case of borderline elevated values, *false-positive results* due to an inappropriate preanalytical preparation, testing method, CMN-metabolism-interfering medication, intense physical stress, severe illness or laboratory error must be considered, and the test shall be repeated upon condition optimization [64].

In patients with PPGLs, MN levels are generally greater than CMNs (A/NA/D) due to continuous production and release of MNs by tumor cells. Patients with false-positive results usually have larger increments in CMNs than in plasma-free MNs because of sympathoadrenal activation [67–69].

*The clonidine suppression test* is indicated in inconclusive situations with borderline elevated NMN levels. A persistently increased level and a lack of a decrease in plasma-free NMN (< 40%) 3 h after the administration of clonidine support the diagnosis of PPGL (sensitivity of 100% and specificity of 96%, respectively) [12, 67]*.* Provocative testing (e.g., glucagon) can be dangerous, adds no value to other current testing methods and is not recommended [12, 70].

In case the probability of a tumor diagnosis is low and there are borderline elevated values, intrapatient longitudinal serial assessments can be useful (e.g., retesting patients 6 months later or more), as the disease growth rate is slow in most cases and involves a doubling time of over 2 years [12, 71]. The measurement of urinary fractionated MNs or concomitant measurement of plasma-free MNs and urinary MN, and CgA should be considered as follow-up tests [12, 72]. In the setting of prospective screening in hereditary forms of the disease, even low increased values have to be considered positive.

*False-negative results* are less frequent but can be observed in microscopic asymptomatic tumors, DA-producing tumors and tumors with CMN synthesis and/or metabolism defects (e.g*.,* in a silent *SDHB*^Mut^ subtype, the enzyme that catalyzes the initial and rate-limiting step in CMN biosynthesis is missing). Additional measurements including CgA (after stopping proton pump inhibitors for at least 10 days) and nonspecific neuroendocrine secretory proteins, as well as imaging studies, are strongly indicated in cases of nonfunctional PPGLs, particularly in *SDHB*^Mut^ carriers [69, 73].

The measurement of 3-MT, the main metabolite of DA, should be considered in patients in whom extra-adrenal HNPGLs are strongly suspected despite normal plasma and urinary MN levels or when metastatic disease is suspected. High-elevated levels of plasma 3-MT indicate the need for preoperative staging, if possible, by radionuclide imaging [12, 59, 68, 73].

Recently, the CMN secretion profile has also been related to the recent molecular cluster classification (see Sect. 3*, risk classifications*): *Cluster 1*: pseudohypoxic Krebs cycle-related (10–15%): NE/NMN and DA/3-MT secretion; *VHL/EPAS1*-related (15–20%): NE/NMN; *Cluster 2*: kinase signaling-related (50–60%): E/MN (especially *RET*) or both E/MN and NE/NMN; and *Cluster 3* (5–10%): Wnt signaling-related: E/MN and NE/NMN and chromogranin A [37, 48].

### Imaging studies

The diagnosis of PPGL relies on the imaging identification of an appropriately located mass with consistent clinical and biochemical features. Once the diagnosis is clinically and biochemically confirmed, imaging studies should be performed to localize and stage the tumor [74]. However, PCGLs, particularly PCCs, are sometimes encountered incidentally on imaging procedures performed for other causes [75].

**Fig. 2:**
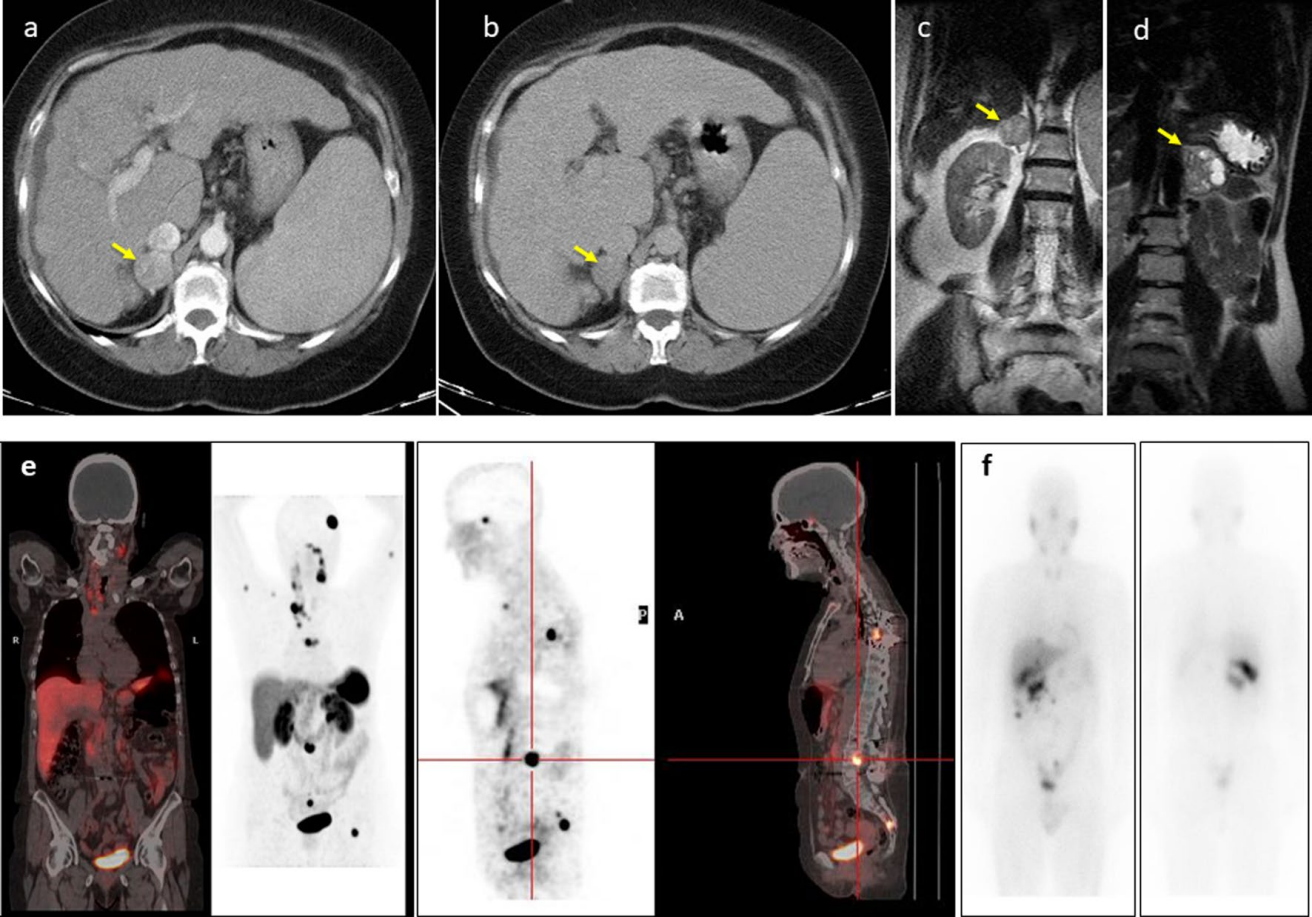
Typical morphological and functional imaging of PPGLs. **a**, **b** Axial contrast-enhanced CT portal (**a**) and delayed phase (**b**) of the upper abdomen showing the pheochromocytoma in the right adrenal gland (yellow arrowhead). Intravenous contrast administration typically enhances avidly due to the capillary-rich framework of the tumor. **c**, **d** Coronal T2-weighted MRI images revealed a homogeneous pheochromocytoma (**c**) in the right adrenal gland (yellow arrowhead), and other pheochromocytomas in the left adrenal gland (yellow arrowhead) with central necrosis are characteristically “light-bulb” bright lesions on T2-weighted imaging (**d**). Pheochromocytomas are potentially malignant (10%), and the only reliable criterion for the diagnosis of malignancy is metastatic spread. **e** A 61-year-old woman with metastatic cervical paraganglioma. 68 Ga-DOTATOC PET/CT study showing bilateral laterocervical lymph nodes, mediastinal involvement and multiple bone metastases. **f** A 56-year-old man was diagnosed with a 44 × 39-mm right adrenal incidentaloma. After right adrenalectomy, a histological study showed pheochromocytoma without evidence of malignancy. Negative genetic study. During follow-up, he presented with recurrence. Body scan with 123-I-MIBG shows lesions in the right renal cell and multiple peritoneal implants, some in contact with the liver surface without being able to rule out secondary infiltration. The patient has received treatment with 131I-MIBG with stabilization of the disease

CT is the most common imaging method used because it is widely available, less expensive and offers better spatial resolution than MRI. PPGLs are usually solid and hypervascular, well-circumscribed masses, ranging from 1 to 15 cm (Fig. 2). Smaller tumors are usually homogeneous, and larger tumors tend to have central necrosis. Some PPGLs can have macroscopic fat simulating adenomas or may have very high attenuation due to hemorrhage or calcifications. There is also a pure cystic form [76, 77].

MRI is not a first-choice imaging tool, but it has the advantage of being free of ionizing radiation and is suitable for children, pregnant women and patients with adverse reactions to iodinated contrast medium. Cystic PPGLs with central necrosis are characteristically “light-bulb” bright lesions on T2-weighted imaging, with low signal intensity at T1. The signal intensity of hemorrhage is predominantly high in T1. If PPGLs contain macroscopic fat, they may also be dark on T2 MR images [78] (Fig. 2).

PPGL cells express different transporters on their surface that allow images to be obtained by different radiotracers depending on the capture mechanisms (NE transporters [^123^I/^131^I- Metaiodobenzylguanidine (MIBG)], type of transporters (glucose transporters (GLUT (^18^F-FDG)), amino acid transporters (^18^F-DOPA) or membrane surface receptors [somatostatin (SST) receptors (^111^In/^99^mTc/^68^ Ga SST analogs)], thus yielding different functional information [79]. The most sensitive functional image for each tumor will depend on the clinical and biochemical profiles and the location of the primary tumor, which are also predictors of the underlying genotype [80].

Positron emission tomography (PET)/CT technology has been shown to be superior to scintigraphy with single photon emission computed tomography (SPECT/CT), with higher spatial resolution, greater sensitivity and fewer indeterminate or equivocal studies [37, 79].

I^123−^MIBG-sensitivity and specificity reach 83–100% and 95–100%, respectively, for the diagnosis of sporadic PCCs. The sensitivity of I^123−^MIBG decreases to 52–75% for the diagnosis of PGLs and to 18–50% for HNPGLs.

^68^Galium (^68^Ga)-DOTA peptide PET showed an overall detection rate of 98.6% in patients with metastatic *SDHB*^Mut^ PPGLs. In HNPGLs, this is considered the functional image of choice. In polycythemia-related PPGLs and in *FH*^Mut^ or *MAX*^Mut^ PPGLs, ^18^F-DOPA PET is the functional imaging of choice, and if not available, I^123−^MIBG-SPECT/CT is recommended [12, 79, 81–83].

Generally, when facing metastatic disease, better results are reported with the use of ^18^F-FDG PET/CT [12, 79, 81, 83]. In these cases, I^123−^MIBG and studies with SST analogs would be reserved for patients with metastatic disease for whom radiometabolic treatment with ^131^I-MIBG and/or ^90^Ytrium (^90^Y)/^177^Lutethium (^177^Lu)-DOTA peptides —peptide receptor radionuclide therapy (PRRT)—is being considered. Recommended PPGL functional imaging studies according to genotype and anatomic location are summarized in Fig. 1.

## Treatment

### Therapeutic strategy

**Fig. 3:**
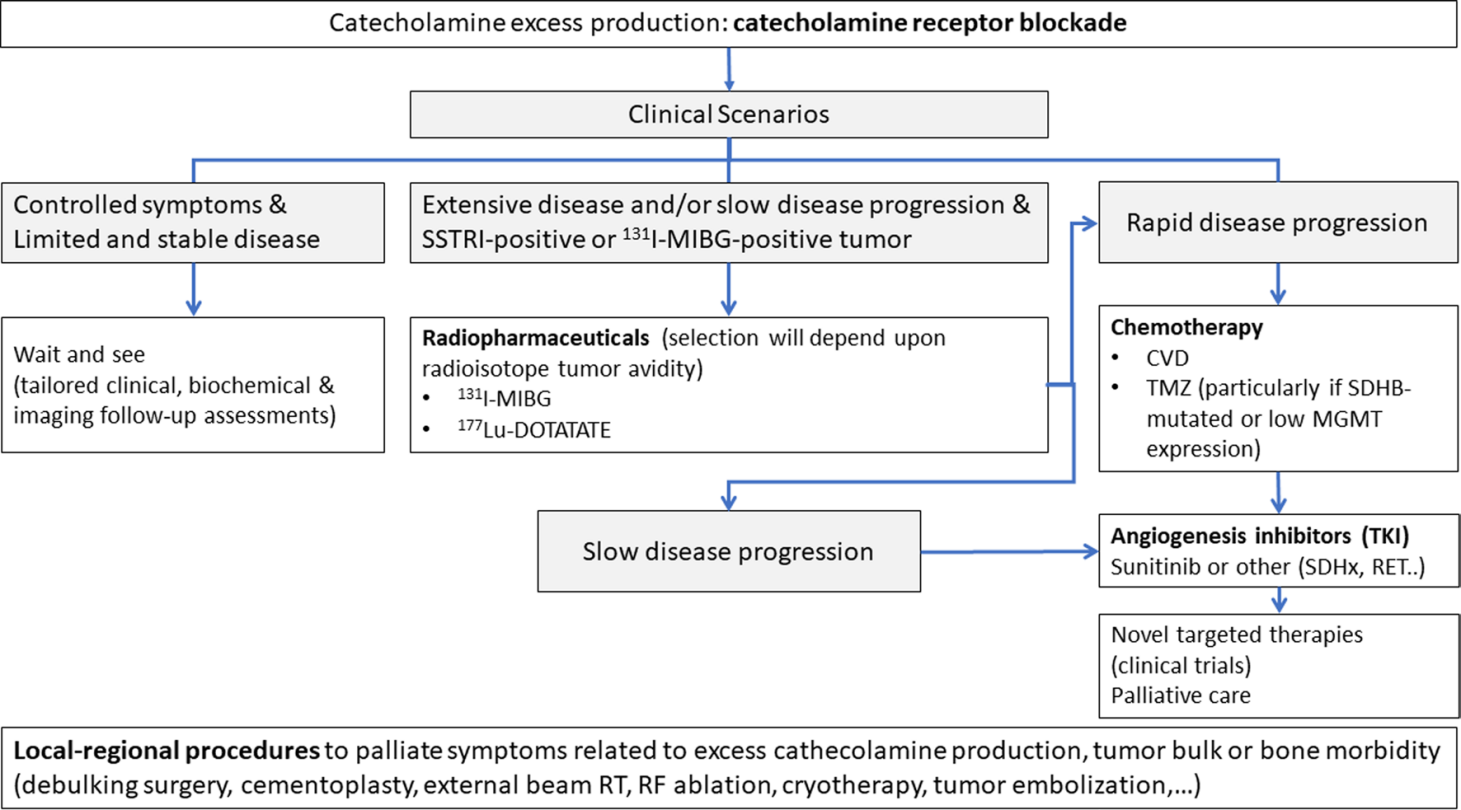
Therapeutic algorithm of metastatic paragangliomas. *CVD*, Cyclophosphamide, Vincristine, Dacarbazine; *MIBG*, 123-I-Metaiodobenzylguanidine; *MGMT*, *O*-methylguanine-DNA methyltransferase; *RF*, radiofrequency; *RT*, radiotherapy; *SDH*, Succinate dehydrogenase; *SSTRI*, somatostatin receptor imaging; *TKI*, Tyrosine kinase inhibitors; *TMZ*, Temozolomide

The therapeutic strategy for PPGLs should be discussed by an expert multidisciplinary team based on patient characteristics (e.g., age, performance status, comorbidities) and tumor features (i.e., primary tumor site, local and distant spread, hormone secretion profile, tumor growth rate, functional imaging and genetic profile). Surgical resection is the mainstay of therapy for the majority of localized PPGLs, with adequate perioperative CMN blockade and cardiovascular monitoring in PCCs and functional PGLs. Adequate timing for surgery and optimal surgical approach are still a matter of debate. Advanced, unresectable disease is not curable, and treatment goals are to slow tumor progression and maintain quality of life. Medical treatment of secretory PPGLs is mandatory to prevent life-threatening events (Fig. 3). Treatment options include watch and wait strategies for indolent tumors, radiometabolic therapy, radiotherapy, chemotherapy, targeted therapy (i.e., antiangiogenic tyrosine kinase inhibitors) and PRRT. Indications and contraindications of these therapeutic options are discussed below, and a therapeutic algorithm is proposed in Fig. 3.

### Catecholamine blockade

Anesthesia, tumor manipulation during surgery, tumor biopsy, adrenal venography and arteriography with ionic contrast can induce excess CMN secretion, hyperadrenergic symptoms and hypertensive crises in patients with PCCs and functional PGLs (FPGLs) [84]. To prevent this potentially fatal phenomenon, the European Endocrine Society Guidelines recommend that patients with PCCs and FPGLs should undergo a 7- to 14-day preoperative preparation with adrenergic receptor blockers as the first choice. Some authors have questioned the universal indication of preoperative α-adrenoceptor blockade given the potential side effects of this therapy, but prospective studies are needed to identify in which patients’ preoperative therapy may be safely avoided. α1 selective blockers (e.g., doxazosin: initiate with 4 mg/day and then titrate up to 16–32 mg/day) are associated with fewer adverse effects, such as reactive tachycardia and sustained postoperative hypotension, compared to nonselective α-blockers (e.g., phenoxybenzamine: initiate with 10 mg/day and then titrate up to 1 mg/kg/day). Calcium channel blockers are the most often used add-on drug class to further improve blood pressure (BP) control. Only after administration of α-adrenergic receptor blockers may a β-adrenergic receptor antagonist (e.g., propranolol or atenolol) be added 2 or 3 days before surgery if the heart rate (HR) exceeds 80/minute (bpm) [85]. Phone or *e*-mail-based preoperative monitoring of BP and HR, orthostatic hypotension, and pharmacological side effects may be implemented in the clinic to avoid multiple visits [86]. Presurgical antihypertensive treatment has been also advised for patients with PPGL and a normal blood pressure[87].

A high-sodium diet and fluid intake are also recommended to reverse CMN-induced blood volume contraction preoperatively and thereby reduce orthostatic hypotension and minimize the risk of severe hypotension after surgical tumor removal [12]. Saline infusion (1–2 L) the evening before surgery is also helpful for this purpose. Esmolol β-adrenergic blockade can be employed during surgery. After surgery, BP and HR monitoring is needed to detect postoperative hypotension requiring vasopressor support. Alpha blockade is not specifically required before iv administration of nonionic contrast media in patients with suspected or known PPGL or related tumors [84].

### Surgery

Surgical resection is the cornerstone of therapy for most localized PPGLs, as it is the only potentially curative therapeutic modality. Careful preoperative planning is required to select the most appropriate surgical technique. This includes precise anatomical characterization of the primary tumor (or tumors if multifocal) location and extension to adjacent structures and/or distant organs and adequate perioperative medical management. The goal of surgery is to achieve complete tumor resection without rupture, including *en bloc* resection of adjacent infiltrated organs if needed.

The complete resection of *HNPGLs*, indicated in young patients, usually requires previous embolization and may be performed in one or more steps depending on the extent of intradural space (IDS) and/or internal carotid artery (ICA) involvement [88]. Elderly or frail patients and those with bilateral multicentric lesions or residual disease may be considered for watch and wait strategies or alternative nonsurgical treatment options [89].

For *cervical and mediastinal PGLs,* a transcervical approach is generally used, while rarely being associated with a transmandibular, transmastoid or infratemporal approach. Carotid body PGLs with ICA involvement have a higher incidence of complications [90]. For *vagal PGLs,* a cervical or posterior tear hole approach is recommended. A 2-stage surgery may be required if there is significant intradural extension. The resection of *jugulotympanic PGLs* [91] has different degrees of complexity. Tympanic PGLs are resected through a low-morbidity transcanal, microscopic/endoscopic approach. Tympanomastoid PGLs implicate transmastoid, transcanal, and infralabyrinthic techniques, occasionally with middle ear removal, that have low morbidity over facial and lower cranial nerves. Jugular PGLs can compromise the ICA and the lower cranial nerves and extend to the IDS. The infratemporal approach employed in these cases entails important technical challenges (protection of the ICA with a *stent* or its occlusion may be required for complete resection) and functional morbidity, particularly if the IDS is involved or the low facial and hypoglossal nerves are affected (> 30%). There is no consensus on the systematic rerouting of the facial nerve. Partial resection may be a valid option in tumors that reach the external auditory canal (EAC) and generate recurrent hemorrhages in elderly patients.

For *abdominal* localized *PPGLs*, a complete surgical resection (PGL) or adrenalectomy (PCC) is indicated. Bilateral adrenal involvement requires bilateral adrenalectomy. Subtotal adrenalectomy with cortical preservation prevents adrenal insufficiency and the need for hormonal supplementation in up to 90% of patients. This procedure is recommended only for cases with a low risk of malignancy, such as MEN2 or VHL syndrome, and not for other genetic syndromes with a greater risk of distant spread or local relapse due to remnant microscopic disease (e.g., *SDHx*^Mut^ or *MAX*^Mut^) [92–94]. In the presence of metastatic disease, total or partial palliative resection may be considered to reduce the disease burden and improve hormonal syndrome control [95].

PCC resection may be open or laparoscopic. Laparoscopic adrenalectomy (LA) is recommended for most PCCs because it is associated with lower morbidity and a shorter postoperative stay than open adrenalectomy (OA). Recurrence rates do not differ between the 2 surgical approaches, with a rate of conversion to OA between 5 and 12% [96]. Minimal tumor manipulation is recommended to avoid excessive CMN release, and anesthetists must also be aware that adrenal vein ligation may induce sudden hypotension. Potential hemodynamic instability is not a contraindication for LA, and the time when the adrenal vein is ligated does not seem to be relevant [92, 96, 97]. LA can be performed transabdominally or retroperitoneally. Both LA approaches achieve adequate resection and have minimal morbidity, with no clear hemodynamic benefits of one over the other. The retroperitoneal approach is especially favorable for simultaneous bilateral adrenalectomy [98, 99]. OA is indicated in bulky (> 6–8 cm) PCCs or in PCCs with a high suspicion of malignancy and/or involvement of neighboring organs or complex locations [92].

*PGL* resection may be more challenging because such tumors are usually located in complex sites (e.g., retroperitoneum, paravertebral, para-aortic in the Zuckerkandl organ and along the inferior hypogastric plexuses adjacent to the urogenital organs) and have a higher risk of malignancy and recurrence. Therefore, an open approach is generally recommended. Non-infiltrative PGLs in favorable locations can be resected by endoscopic surgery [98].

Acute postoperative complications, such as hemodynamic and metabolic instability with hyper- or hypotension and hypoglycemia, can be avoided with appropriate CMN blockade and fluid replacement.

Acute and chronic adrenal insufficiency should be assessed, and hormonal replacement therapy should be appropriately administered in bilateral total and cortical sparing adrenalectomy or unilateral cortical sparing adrenalectomy of a sole remaining adrenal gland.

### Radiotherapy and radiometabolic therapy

#### Radiotherapy

The greatest experience of PGL radiotherapy (RT) comes from the treatment of glomus jugular tumors, as RT constitutes a noninvasive therapeutic option that is appropriate in locations with high surgical risk or when patients are not candidates for surgery (patients with carotid or intracranial involvement) [100].

Conventional RT achieved modest responses (20–30%) that were surpassed by radiosurgical techniques administered in single doses (12–15 Gy) and, thereafter, by stereotaxic ablative radiotherapy (SABR), with doses of 20–25 Gy in 3–5 fractions, leading to tumor control rates of 90–100% and symptomatic improvement in 80% of patients [101].

#### Radiometabolic therapy

Systemic radiometabolic treatment is an option for disease control in patients with inoperable locally advanced or metastatic disease and documented tumor uptake of the corresponding radioisotope. It is generally considered for symptomatic patients with slow-growing tumors and significant tumor volume or disease progression [102, 103].

Functional imaging studies should be carried out with ^123^I-MIBG and/or radiolabeled SST analogs to assess the affinity of the tumor for the radiotracer and to choose the most appropriate radiopharmaceutical for each case before peptide receptor radionuclide therapy (PRRT) [104].

The largest accumulated experience with ^123^I-MIBG shows that over 50% of patients with mPPGL are candidates for radiometabolic treatment. There is no consensus regarding the preferred treatment protocol, optimal dose and time interval between doses, or response criteria. With the administration of a medium–high activity (200–275 mCi) of ^123^I-MIBG, repeated every 3 months depending on the achieved response, objective responses have been documented in 30–60% of cases, a hormonal response in 10–71% of patients and a symptomatic response in 23–90% of patients [105, 106]. The main side effects are related to cumulative dose-dependent bone marrow toxicity and renal toxicity. Thyroid radiotracer uptake should be blocked prior to therapy to prevent hypothyroidism, and blood counts and renal function should be monitored.

More recently, a novel ^123^I-MIBG derivative has been developed, Iobenguane ^131^I or high-specific-activity (HSA)- ^123^I-MIBG, produced from a solid-phase ultratrace precursor that eliminates the presence of cold MIBG and is able to deliver a high radioactivity level per dose (~ 2500 mCi/mg; 92.5 MBq/μg). With conventional ^123^I-MIBG cold MIBG competes with radiolabeled MIBG for the NE transporter, reducing labeled MIBG uptake by the tumor cell, thus limiting efficacy and increasing the levels of circulating NE that can lead to life-threatening acute hypertensive crisis during or shortly after drug administration. A phase 2 trial showed that 17 of 68 PPGL patients (25%) treated with HSA-^123^I-MIBG had a durable reduction in baseline antihypertensive medication use, and 92% achieved a partial response or stable disease as the best objective response within 12 months. The median OS was 36.7 months, and no patients had drug-related acute hypertensive events. Based on these data, HAS-^123^I-MIBG received FDA breakthrough therapy designation and was approved in July 2018 for the treatment of patients with iobenguane scan-positive, advanced or mPPGLs who require systemic anticancer therapy [107].

Experience in the use of PRRT for the treatment of PPGLs is limited, although early results look promising. Radiological control has been described in 80% of patients with metastatic *SDHB*^Mut^ PPGL treated with ^177^Lu-DOTATATE [108, 109]. However, prospective studies are necessary to determine the role of PRRT in the control of patients with inoperable advanced or mPPGLs.

### Systemic therapy

Metastatic disease, unless amenable to complete surgical resection, is incurable. Systemic treatment options are limited but can offer symptom palliation and disease control. However, due to the relatively indolent nature of PPGLs, these therapies are generally reserved for patients with clear disease progression or severe symptoms caused by hormone secretion or mass effects. Evidence to support treatment decisions is poor, although increasing data suggest that different molecular subtypes driven by distinctive oncogenic pathways may have unique sensitivity profiles to specific drugs [49, 110]. International collaborative efforts are key to make adequately sized prospective trials feasible.

Chemotherapy is considered the treatment of choice for patients with advanced PPGLs who have progressed to or are not suitable candidates for MIBG or PRRT. Cyclophosphamide, vincristine and dacarbazine (CVD) chemotherapy is the most widely used regimen and is considered the standard of care despite the lack of prospective trials [49, 111, 112]. A systematic review of four retrospective series that included 50 patients reported an objective tumor response rate of 41% (4% complete and 37% partial responses) and a biochemical response rate of 54% (14% complete) [112]. Two of these studies reported median durations of response of 20 and 40 months, respectively. In the largest single-institution experience with chemotherapy (54 patients), 33% of patients achieved a response, defined as improved BP control and/or reduced tumor size. OS was 6.4 years for responders vs 3.7 years for non-responders, a difference that was statistically significant in multivariate analysis [111]. The most common toxicities include myelosuppression, peripheral neuropathy and gastrointestinal toxicity, which may occasionally be severe but are generally transient and manageable. A retrospective study of 15 patients treated with temozolomide (150–200 mg/m^2^/day d1–5 q28 days), 8 of whom had received prior chemotherapy, documented 5 partial responses (33%) that occurred only in patients with *SDHB* mutations [113]. The median progression-free survival (PFS) was 13.3 months (19.7 vs 2.9 months in *SDHB*^Mut^ vs noncarriers). *SDHB*-germline mutations were associated with O-methylguanine-DNA methyltransferase (*MGMT*) promoter hypermethylation and low MGMT protein expression in a cohort of 190 samples of the French national PPGL network [113]. These findings suggest that *MGMT* epigenetic silencing in *SDHB*^Mut^ carriers may render them particularly sensitive to this alkylating agent. Successful outcomes have also been reported in two patients with *SDHB*^Mut^ metastic PGLs treated with temozolomide metronomic schedules (75 mg/m^2^/day × 21/28 days) following progression to prior CVD therapy [114]. More recently, an increased activity in the Poly(ADP-ribose)polymerase (PARP) DNA repair system has been described in *SDHB*^Mut^ PPGLs, associated with chemo-resistance[115]. The PARP-inhibitor olaparib was shown to markedly potentiate the therapeutic effect of TMZ with prolonged overall survival of mice with *SDHB* knockdown PPGL allograft [115]. Based on these findings, a trial investigating the synergistic effect of the addition Olaparib to TMZ is currently undergoing (NCT04394858).

A number of tyrosine kinase inhibitors (TKIs) are being explored due to the key role that angiogenesis regulation plays in PPGLs, particularly in Cluster 1 (*SDH*- and *VHL*-driven PPGLs) and some Cluster 2 tumors (i.e., *RET*). The phase II SNIPP trial evaluated sunitinib in 25 patients with progressive PPGLs [116]. The overall response rate was low (13%) in the overall unselected population, although all three partial responses occurred in patients with germline mutations in *SDHA*, *SDHB* and *RET* (with this last patient remaining on treatment 7 years later). The disease control rate (DCR) was 83%, meeting the study primary endpoint, and the median PFS was 13.4 months. The most common severe side effects were fatigue and thrombocytopenia (16% each), and three patients discontinued treatment due to cardiovascular adverse events. Sunitinib is currently being assessed in the first randomized, placebo-controlled trial ever conducted in PPGLs, the FIRSTMAPPP trial. A phase II trial with pazopanib was terminated early due to poor patient accrual. One of the six evaluable patients achieved a partial response (17%), and the median PFS and OS periods were 6.5 and 14.8 months, respectively [117]. Similarly, preliminary data of a phase II trial with axitinib reported an objective response in three of nine treated patients (33%) and some degree of tumor shrinkage that did not qualify for partial response in five additional patients, which was associated with biochemical response [118]. Other TKIs (cabozantinib, lenvatinib, etc.) are currently being evaluated in clinical trials (https://clinicaltrials.gov/).

Finally, some other drugs active in the treatment of NETs, such as ‘cold’ SST analogs and interferon, have been poorly addressed in this setting although they are also used for the treatment of PPGLs [114, 119]. Currently, a phase II prospective trial is assessing the role of the SST lanreotide in patients with advanced disease (NCT03946527).

### Local ablative therapy and supportive care

In patients with progressive advanced or mPPGLs, the treatment goals are to manage hormone-related symptoms, control tumor growth and prolong OS. The use of local ablative therapies in this setting can improve local control and palliate symptoms [120]. The indication must be individualized and discussed within the multidisciplinary team and carefully balanced versus other treatment options for patients with mPPGLs.

There is no prospective study to assess differences in outcome for patients receiving different ablative treatments for advanced disease. However, two recent retrospective studies published by the Mayo Clinic have analyzed the outcome of patients with mPPGLs receiving local therapies. The first study showed median OS and PFS rates of 24.6 and 33.7 years, respectively, at a median follow-up of 8.2 years (range, 0.01 to 54.1 years). Among the 272 patients analyzed, 97% underwent additional surgical resection (for primary tumors or metastases). In addition, palliative RT, radiofrequency ablation, embolization procedures, stereotactic radiotherapy, cryoablation and percutaneous ethanol injection were performed in 47%, 9%, 8.8%, 5.8%, 4.7% and 2% of the patients, respectively. Almost half of the patients (45%) survived > 10 years [121]. The second study reported the efficacy and safety of radiofrequency ablation, cryoablation and percutaneous ethanol injection in these patients. Radiographic local control was achieved in 69/80 (86%) lesions. Improvement in metastasis-related pain or symptoms of CMN excess was achieved in 12/13 (92%) patients. Thirty-three (67%) procedures had no reported complications [120].

PPGLs are among the solid tumors that most frequently spread to the skeleton and cause skeletal-related events (SRE) (i.e. pain, bone fracture, and spinal cord compression are commonly the first manifestation of metastatic disease (31%). SREs should, therefore, be properly addressed, as they compromise survival and can seriously impair patients’ quality of life. A multidisciplinary approach with specialists in endocrinology, oncology, palliative care, radiotherapy, orthopedic surgery and neurosurgery is of utmost importance.

## Follow-up recommendations

*Short-term postoperative follow-up* should include clinical and biochemical evaluation (MNs, 3MT/CgA if other markers are negative) 2–6 weeks after recovery. Imaging is recommended 3 months after recovery in patients with persistent postoperatively altered biochemical markers, silent PPGLs and absence of preoperative biochemical evaluation [122].

*Long-term follow-up* is mandatory in all patients, as they are all considered at risk of tumor recurrence, and the clinical behavior of PPGLs is remarkably variable, especially in PPGLs associated with hereditary syndromes. Ten-year follow-up is recommended for all patients with resected PPGLs and lifelong personalized follow-up for patients with hereditary forms of the disease; such follow-up should be performed by a multidisciplinary team at a tertiary center whenever possible. Whereas in some patients with mPPGLs, the discovery of metastases may precede the discovery of the primary tumor, others may develop metastases many years after the initial diagnosis [121, 123].

Candidates for intensified surveillance have to be identified. Male sex, older age at primary tumor diagnosis (≥ 76 years), larger tumor size (> 4.5–5 cm), failure to undergo complete surgical resection of the primary tumor, DA hypersecretion and synchronous metastases are associated with shorter survival ^15–21^. Tumor size, extra-adrenal location and germline *SDHB* mutations are independent risk factors for mPPGLs [13, 60, 61, 124–126].

Currently, no specific follow-up protocols are established. The frequency of surveillance should be based on a number of factors, such as the affected gene, genotype–phenotype correlation, symptomatic or silent pattern of the disease, potential severity of the disease, penetrance and family history. Overall, it is recommended to carry out annual clinical anamnesis, physical exam (including blood pressure control) and biochemical monitoring (MNs, ± 3-MT and optional CgA in MNs/3-MT negative PPGLs). Imaging studies are recommended yearly in suspected cases (based on clinical or biochemical evaluation) or every 2–3 years in silent PPGLs. To avoid cumulative irradiation, body or head/neck MRI should be considered the imaging procedure of choice for surveillance, especially in children and during pregnancy, reserving CT and functional NM imaging to characterize pathological findings in cases of relapse. Specific monitoring of the other diseases associated with each syndrome should also be performed [12, 122].

Regarding the role of functional imaging during follow-up, experts [12] recommend the use of ^123^I-MIBG scintigraphy when the risk of metastasis or disease recurrence is high, and ^18^F-FDG PET/CT is only indicated in established metastatic disease. The use of more than one functional imaging modality may be considered in selected cases, such as the use of both ^68^ Ga-DOTATATE and ^18^F-FDG PET/CT in patients with small lesions when there is a high likelihood of metastatic disease and in *SDHx*^Mut^ patients [37, 127].

*Follow-up of asymptomatic carriers* There is no sufficient clinical epidemiological evidence from clinical data to perform general recommendations for surveillance. The main aim of surveillance programs in healthy mutation carriers, especially in SDHX-mutation carriers, is to identify disease at an early stage in order to allow a successful intervention at the appropriate time, improve cure rates and limit the chance of malignant transformation and metastasis. Modality and frequency of screening that individual centers adopt will be dependent on local expertise, availability and costs. The appropriate age to start screening will vary according to the specific hereditary syndrome including the malignant and metastatic potential associated with the identified genetic mutation. In children it is generally recommended between 5 and 10 years of age or 5 years before the youngest clinical manifestation in the family. Biochemical and clinical monitoring follows diagnosis recommendations mentioned above. Debate is ongoing regarding the frequency and type of the functional image probe to be done. Most tumors are diagnosed in the first screening image performed. The Endocrine Society Guidelines [12] emphasized that consideration for any imaging modality requires prior positive clinical or biochemical evidence of disease, except in case of a personal or family history of HNPGL related or not to a hereditary form. More recent studies and meta-analysis report the need of periodical image evaluation, the recommended frequency varying generally between 2 and 3 years [47, 128–132]. Currently, translational research stratification scores have been developed to estimate the risk of new PPGL events and the frequency of metastatic disease [133]; however, evidence from longitudinal studies is still needed, and guidelines for follow-up continue to evolve. National and international registries are fundamental to collect information necessary to deliver updates that permit the elaboration of clinical guidelines.
